# Using DNA Metabarcoding To Evaluate the Plant Component of Human Diets: a Proof of Concept

**DOI:** 10.1128/mSystems.00458-19

**Published:** 2019-10-08

**Authors:** Aspen T. Reese, Tyler R. Kartzinel, Brianna L. Petrone, Peter J. Turnbaugh, Robert M. Pringle, Lawrence A. David

**Affiliations:** a Society of Fellows, Harvard University, Cambridge, Massachusetts, USA; b Department of Ecology and Evolutionary Biology, Brown University, Providence, Rhode Island, USA; c Medical Scientist Training Program, Duke University, Durham, North Carolina, USA; d Department of Molecular Genetics and Microbiology, Duke University, Durham, North Carolina, USA; e Department of Microbiology and Immunology, University of California San Francisco, San Francisco, California, USA; f Department of Ecology and Evolutionary Biology, Princeton University, Princeton, New Jersey, USA; g Center for Genomic and Computational Biology, Duke University, Durham, North Carolina, USA; University of Trento

**Keywords:** human diet, DNA metabarcoding, *trn*L(UAA)-P6, diet log

## Abstract

Dietary intake is difficult to measure reliably in humans because approaches typically rely on self-reporting, which can be incomplete and biased. In field studies of animals, DNA sequencing-based approaches such as metabarcoding have been developed to characterize diets, but such approaches have not previously been widely applied to humans. Here, we present data derived from sequencing of a chloroplast DNA marker (the P6 loop of the *trn*L [UAA] intron) in stool samples collected from 11 individuals consuming both controlled and freely selected diets. The DNA metabarcoding strategy resulted in successful PCR amplification in about 50% of samples, which increased to a 70% success rate in samples from individuals eating a controlled plant-rich diet. Detection of plant taxa among sequenced samples yielded a recall of 0.86 and a precision of 0.55 compared to a written diet record during controlled feeding of plant-based foods. The majority of sequenced plant DNA matched common human food plants, including grains, vegetables, fruits, and herbs prepared both cooked and uncooked. Moreover, DNA metabarcoding data were sufficient to distinguish between baseline and treatment diet arms of the study. Still, the relatively high PCR failure rate and an inability to distinguish some dietary plants at the sequence level using the *trn*L-P6 marker suggest that future methodological refinements are necessary. Overall, our results suggest that DNA metabarcoding provides a promising new method for tracking human plant intake and that similar approaches could be used to characterize the animal and fungal components of our omnivorous diets.

**IMPORTANCE** Current methods for capturing human dietary patterns typically rely on individual recall and as such are subject to the limitations of human memory. DNA sequencing-based approaches, frequently used for profiling nonhuman diets, do not suffer from the same limitations. Here, we used metabarcoding to broadly characterize the plant portion of human diets for the first time. The majority of sequences corresponded to known human foods, including all but one foodstuff included in an experimental plant-rich diet. Metabarcoding could distinguish between experimental diets and matched individual diet records from controlled settings with high accuracy. Because this method is independent of survey language and timing, it could also be applied to geographically and culturally disparate human populations, as well as in retrospective studies involving banked human stool.

## Abstract (precis)

Current methods for capturing human dietary patterns typically rely on individual recall and as such are subject to the limitations of human memory. DNA sequencing-based approaches, frequently used for profiling nonhuman diets, do not suffer from the same limitations. Here, we used metabarcoding to broadly characterize the plant portion of human diets for the first time. The majority of sequences corresponded to known human foods, including all but one foodstuff included in an experimental plant-rich diet. Metabarcoding could distinguish between experimental diets and matched individual diet records from controlled settings with high accuracy. Because this method is independent of survey language and timing, it could also be applied to geographically and culturally disparate human populations, as well as in retrospective studies involving banked human stool.

## INTRODUCTION

Reliable dietary data are needed for human biomedical research and for developing appropriate nutritional recommendations. Methods of diet tracking in both research and clinical contexts frequently depend on self-reporting, whether in the form of diaries in which meals are logged (diet records), prompts to remember foods eaten in the past day (24-h recalls), or surveys that ask individuals to summarize their eating habits over time frames of up to a year (food-frequency questionnaires) (1). However, such human diet assessments have notoriously low accuracy due in part to inaccuracies and bias associated with human memory (2–4). These methods can be so misleading that the majority of diet surveys have been found to routinely misreport caloric intake (2). Furthermore, a greater degree of nutrition education did not improve—indeed, worsened—the accuracy of self-reported diet information (5). Even if diet items are accurately reported, accounts typically lack abundance data (i.e., logs note whether an ingredient was present in the diet but not the amount consumed), and thus, self-reported data are likely to overestimate the importance of rare food items and underestimate common ones. There is therefore a need for alternative methods of quantifying human diet composition (4).

DNA sequencing methods are increasingly used to infer the diets of wild animal populations for which reliable observational data are difficult or impossible to obtain (6). An amplicon-based sequencing technique, known as DNA metabarcoding, is commonly applied in zoology (7–9), microbial community ecology (10), and environmental DNA studies (11) to identify species based on reference databases containing diagnostic sequences (DNA barcodes). Sequencing of plant biomarkers has been used to assess the diet composition of individual herbivore and omnivore species (6, 12–14), to compare diets across species and analyze food web networks (8, 15, 16), and to evaluate differences in food selection by model lab mice under experimentally controlled conditions of nutrient and disease stress (17). Importantly, there is clear potential to apply similar techniques to characterize human diet composition in ways that may support biomedical research and applications (7).

We investigated the utility of DNA metabarcoding for characterizing the plant component of human diets. We applied to human stool samples a widely used protocol for plant DNA metabarcoding, based on amplification and sequencing of the *trn*L(UAA)-P6 marker from chloroplast DNA (6, 11). This marker is useful for dietary analysis due to its short length, conserved primer sites, and interspecific variation (6, 7). It has previously been shown to successfully identify plant DNA in human feces (7) and used to analyze the diet composition of wild herbivores (8, 11, 12, 18). We analyzed samples from a previous diet-intervention study (19) to investigate if (i) self-reported differences in diet composition correspond to DNA-based differences in diet composition and (ii) DNA-based methods can identify experimentally induced dietary changes in diet composition.

## RESULTS

We applied DNA metabarcoding to fecal samples from a cohort of 11 individuals who consumed prepared diets with controlled sets of plant ingredients (19). During the study, participants were fed two controlled diets with free eating during a preceding baseline and following washout periods: the plant diet arm included selected grains, legumes, fruits, and vegetables while the animal arm included prepared meats, eggs, and cheeses. We analyzed samples from the end of each diet intervention as well as various free-eating time points (see Fig. S1 in the supplemental material).

10.1128/mSystems.00458-19.1 FIG S1 Diet study design and sampling windows (black bracketed lines) for DNA metabarcoding analysis. Successfully sequenced sample sizes are specified by day. (Adapted from reference 19 with permission of the publisher.) Download FIG S1, PDF file, 0.7 MB. Copyright © 2019 Reese et al. 2019 Reese et al. This content is distributed under the terms of the Creative Commons Attribution 4.0 International license.

In total, we observed a PCR band in 50% of the 54 human samples available from the prepared-diet study. Success varied significantly by diet type (*P* = 0.05, χ^2^ = 2.83, DF = 1, chi-square test), with more samples that were collected during the animal diet arm failing to amplify (71%) than those from the plant diet arm (30%). Approximately half of the baseline and washout samples (48%) were successful. From the PCR-positive samples, we obtained 2,113,660 *trn*L-P6 sequence reads that perfectly matched 78 sequences from the reference database. After combining sequences that could not be fully distinguished at the species level (see Materials and Methods), our analyses captured 47 dietary plant taxa. Of these, 39 were identifiable to species level, 4 were identifiable to genus level, and 4 included multiple genera (Table S3). These perfectly matched sequences represented over 70% of the total sequence reads. The median perfect-match read depth was 4,273 per sequence taxon (range = 1 to 556,223).

**FIG 1:**
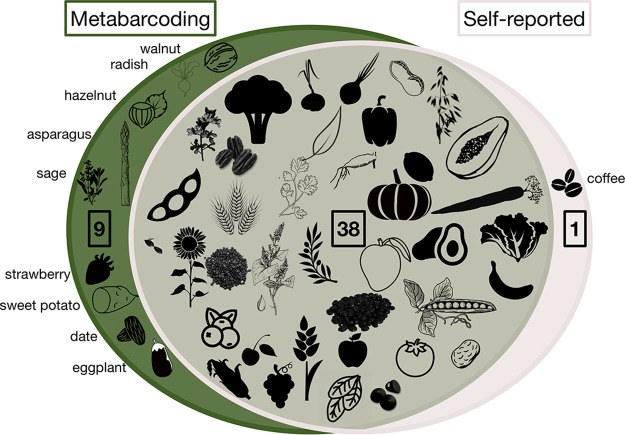
Most plant taxa (79%) were recorded as present at least once in both diet diaries and metabarcoding. Whereas some plants (19%) were found via metabarcoding but not recorded in diaries, only one (coffee) was recorded in diet diaries but absent in metabarcoding. Common names of taxa unique to one method are specified around the Venn diagram.

We compared DNA metabarcoding results to diet diaries kept by participants before, during, and after the controlled-feeding study and found that 38 taxa (79%) appeared in both the sequencing and diary data sets, whereas only one (2%) was solely recorded in the diet diaries (Fig. 1). We next calculated the percentages of plant taxa recorded by participants as having been consumed that were captured by DNA metabarcoding (recall) and the percentage of plant taxa detected by DNA metabarcoding that was also reported in diet diaries (precision). High recall would suggest that metabarcoding yields data that are similar to self-reports. Low precision is harder to interpret, as it could indicate that metabarcoding captured aspects of diet that diaries did not and/or that some proportion of the sequences are false positives. Across all fecal samples, the metabarcoding method had a recall of 0.76 and precision of 0.26 for determining presence/absence of dietary plants in light of the participant's diet record; these two measures are summarized by an F-measure of 0.39 (Table S5). Recall, precision, and F-measure all range from 0 to 1, with 1 representing perfect performance; the F-measure calculated here is unweighted (i.e., assigns equal importance to recall and precision) and is the harmonic mean of recall and precision, which means it tends toward the lesser value of the two. We observed elevated rates of putative false positives for some plants: 25 taxa had false-positive rates greater than 50%.

**FIG 2:**
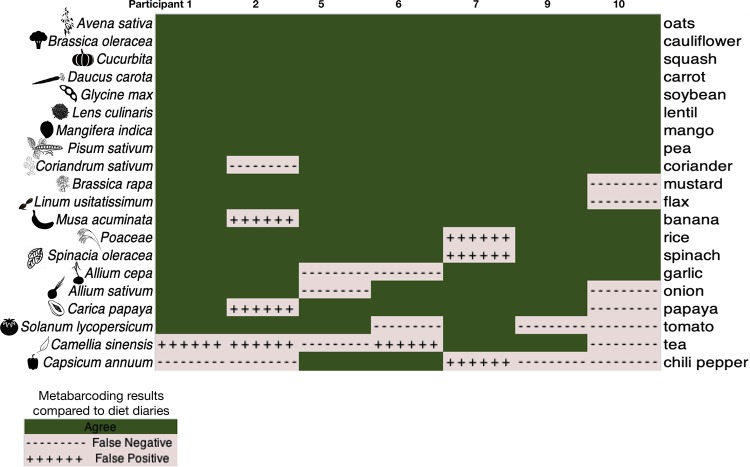
Congruence (green) between diet-diary entries from the day preceding sampling and metabarcoding was common for controlled diet ingredients during the plant-diet arm. Disagreement between metabarcoding data and the dietary diary, either false negative or false positive, is indicated in pink. Latin names of foods are presented to the left of the heat map, and common names are given on the right.

In fecal samples from the plant-diet arm alone, recall, precision, and F-measure were greater than for the complete data set—0.86, 0.55, and 0.67, respectively (Fig. 2; Table S5). This difference is unsurprising because self-reports are expected to be more accurate during this period of controlled, limited diets and there is also likely higher plant DNA content in stool samples. The only plant-based food present in diet logs that was never detected by metabarcoding was coffee, whereas plants that were inconsistently detected included tea and peppers—in general, beverages and spices may be hard to detect due to low abundance in the diet and high rates of processing.

Coarsening the taxonomic resolution of plant identifications marginally increased the apparent recall of the DNA metabarcoding method (0.73 at species level versus 0.82 at family level) as well as its precision (0.25 versus 0.33, respectively), reflected in an improvement in F-measure (0.37 versus 0.47; Table S5). This was also the case in the plant-diet arm-only samples (recall 0.84 at species level versus 0.92 at family level, precision 0.59 versus 0.59, and F-measure 0.69 versus 0.72). Precision and recall are inversely related; the increase in both metrics that we observed here occurs because the plant taxa involved in the comparison change (in both number and detection status) when they are aggregated to a higher taxonomic level.

We did observe the expected inverse relationship between metrics when the underlying plant taxa remained the same and the detection threshold was varied. Requiring a sequence to exceed a count threshold of 1% or 5% of total reads in order to be defined as present in a given sample led to substantial improvements in precision (increases to 0.51 and 0.51, respectively) but at the cost of recall (decreasing to 0.34 and 0.17, respectively; Table S5). Combined evaluation of these two parameters in the F-measure showed an overall improvement in performance at the 1% threshold (0.41) but a deterioration at 5% (0.25). Interestingly, this trend was not replicated when considering samples from the plant-diet arm only, for which F-measure consistently decreased with an increasing read threshold (to 0.45 at 1% and 0.26 at 5%; derived from recall of 0.30 versus 0.15 and precision of 0.90 versus 0.85 at the 1% and 5% levels, respectively; Table S5). This contrast suggests that imposing a read threshold on the plant-only samples filters out more true positives than false positives and leads to an overall decrease in performance, while a modest read threshold applied to samples including those from nonintervention periods has the opposite effect. This supports the notion that missed reporting of trace plants in diaries but detection by metabarcoding (deemed “false positives” in our analysis framework) has a more prominent effect in freely eaten diets, which included a larger variety of prepared and processed foods that may have obscured these ingredients from the consumer. By comparison, in the plant diet arm, all such diet components were known and could be exhaustively coded from a simply reported menu item (e.g., “Dinner curry”) by investigators. Finally, the striking decrease in recall observed in the plant-diet arm samples by applying a 1% read threshold (from 0.86 to 0.30) indicates that true positives are being filtered from the comparison to diet records at this threshold and, thus, that not all low-abundance DNA metabarcoding reads represent false positives.

**FIG 3:**
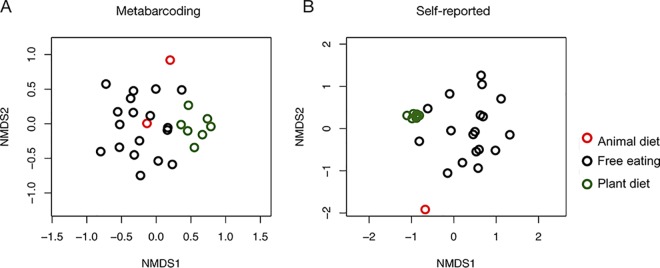
Nonmetric multidimensional scaling (NMDS) of metabarcoding (A) and diet diaries (B) shows separation between experimental diet arms. Samples from participants during the free-eating periods are shown in black (*n* = 18), those from the plant-rich diet period are shown in green (*n* = 7), and those from the animal-rich diet period are shown in red (*n* = 2).

DNA metabarcoding and diary-based methods for characterizing participants’ plant intake yielded similar—but nonidentical—results. There was a positive, but weak, correlation between Bray-Curtis dissimilarity of metabarcoding results and data from participant diaries (Mantel statistic = 0.28, *P = *0.002). We also found that DNA-based dietary composition differed significantly between baseline and experimental diet stages (permutational multivariate analysis of variance [PERMANOVA]: *P < *0.001, *R*^2^ = 0.19, DF = 3, pseudo-F value = 1.80), as visualized using nonmetric multidimensional scaling (NMDS) (Fig. 3). Diary-reported diet composition also differed significantly as a function of experimental diet stage (PERMANOVA: *P < *0.001, *R*^2^ = 0.37, DF = 3, pseudo-F value = 4.58). The two animal-diet samples that we succeeded in amplifying were nearly entirely dissimilar from the plant-diet samples (Bray-Curtis dissimilarity = 0.99 ± 0.01), consistent with the experimental design.

Last, we tested whether differences in plant intake measured by DNA metabarcoding were associated with overall patterns in gut microbial composition or metabolism. We calculated Bray-Curtis dissimilarity matrices based on bacterial relative abundance measured with 16S rRNA gene amplicon sequencing and fecal short-chain fatty acid concentrations (a measure of microbial metabolic functioning) for the baseline samples, during the plant-diet intervention, and during the washout period. Microbial composition was not significantly correlated with either diet self-reports or metabarcoding results at any time point (Mantel tests, *P > *0.05). Similarly, we did not detect associations between either method of diet analysis and short-chain fatty acid concentrations (Mantel tests, *P > *0.05). These nonsignificant results may reflect the relative homogeneity of food intake profiles between participants in the plant-diet intervention.

## DISCUSSION

We have shown that dietary plant DNA can be amplified and sequenced from human stool using methods commonly applied to wildlife studies. Plant DNA could identify and distinguish experimental and noninterventional diet compositions based on plant taxa commonly consumed by humans. As we were able to detect human consumption of 47 unique plant taxa encompassing 29 plant families, 39 genera, and 39 species, and DNA metabarcoding has previously been employed to characterize the diets of diverse herbivores and omnivores in the wild (8, 13), we believe this approach could be applied effectively to more geographically and culturally disparate human populations in the future.

Before this method is ready for widespread application in biomedical research, further methodological refinements will be necessary. A potentially limited ability to characterize diet composition of free-feeding humans is a challenge that will need to be overcome, because it will often be impossible to distinguish between errors arising from metabarcoding or diet diaries if the two data sources are in conflict outside experimentally controlled conditions. Both potential sources of error could contribute to the imperfect precision and recall documented here, especially during the free-feeding period of the study. As such, our measurements may best be considered estimates rather than exact precision and recall, as perfect knowledge of participant’s diets was unavailable.

Improving the accuracy of human diet diaries may continue to be challenging, owing to the inherent imperfection of memory, but improvements to dietary DNA metabarcoding strategies are occurring rapidly (20, 21). First, many recent improvements to DNA metabarcoding strategies focus on overcoming technical challenges, including optimizing sample handling and extraction, overcoming potential PCR biases, and developing computer algorithms that can more effectively detect and remove aberrant DNA sequences (20, 22, 23). Although our protocol relied on methods that were state of the art at the time, researchers should carefully consider the most recent developments when applying this approach in the future. In particular, further DNA-cleaning protocols to remove polyphenols and other PCR inhibitors commonly found in plants could reduce the rate of PCR failures. Second, researchers are focusing on important considerations related to study design: it is challenging to obtain a highly precise dietary profile from a single sample, and studies pursuing this goal may require a high degree of technical replicates (replicated DNA extractions and PCRs) (24); yet, experimental and computer-simulation analyses suggest that population-level analyses based on well-designed DNA metabarcoding studies can support robust dietary comparisons except in cases of extreme primer bias for or against the most abundant “true” dietary item (20). Despite these potential study limitations, our analysis revealed the expected pattern of nearly complete dietary differentiation between experimental populations that were fed plant- and animal-based diets (Bray-Curtis = 0.99), even with a relatively small sample size (*n* = 27) (Fig. 3).

Important aspects of human physiology and diet composition will be important to consider in the design of DNA metabarcoding experiments that involve people. Diet composition affects gut retention time (25–28), meaning that fecal samples collected simultaneously from two individuals do not necessarily contain foods that the two individuals consumed at the same point in time. DNA copy numbers in fecal samples may also be biased due to differential DNA content in the tissues eaten, digestion of DNA in the gut, and/or recovery of DNA from the resulting specimen. In order to overcome the challenge of discerning how much error exists in the DNA-based analyses and diary-based summaries, future studies should examine large cohorts of people consuming controlled, but varied, diets over time. Although we found DNA from cooked plant material in feces, food preparation and processing could also affect the digestibility of plants (29) and may degrade DNA itself. Notably, coffee—the only plant-based food that was recorded in diaries but never detected by DNA metabarcoding—is derived from seeds that are first roasted and then steeped at high temperatures, all of which could contribute to low quantity and quality of chloroplast DNA markers. Future work should assess how the abundance of DNA markers in feces is impacted by cooking technique and the type of plant tissue consumed. Last, humans consume primarily domesticated plants: only 15 crop species provide almost 70% of the world’s calories (30). For example, cruciferous vegetables such as broccoli, kale, and cabbage are all the same species (Brassica oleracea) and require extensive sequencing to be distinguished (e.g., 11 to 13 microsatellites) (31, 32); we found here that apples (genus *Malus*) and pears (genus *Pyrus*), as well as rice, rye, and wheat (family Poaceae), are identical at the *trn*L-P6 locus. The use of single-marker loci in DNA metabarcoding studies may therefore be insufficient to differentiate between some foods that are typically considered distinct, including phenotypically and nutritionally variable plants or plant parts, and approaches based on multiple markers warrant exploration. A more diverse reference database would be necessary regardless if this approach were to be applied to human populations who consume more wild plants (33–35).

Despite these current limitations, DNA-based dietary analyses hold promise for tracking human plant intake. In particular, we believe this approach could be used to increase the frequency with which human plant diet is monitored in biomedical research and clinical applications, as metabarcoding complements standard methods in research on digestion and gastrointestinal health. Fecal samples are regularly collected by medical providers as well as by researchers for microbiome analysis but are to our knowledge not used for dietary sequencing in humans. In the future, DNA metabarcoding could enable investigators to retrospectively infer plant and animal intake among study participants who have banked stool samples but not tracked their diets; such samples are increasingly abundant due to the growing number of human gut microbiome studies (36). Here, the same DNA extractions were used for microbial community profiling and plant metabarcoding. Comparisons between these produced results consistent with the previous finding that the plant-diet experimental treatment was associated with only weak changes in microbiota structure (19). Other applications might include assessing compliance during dietary intervention studies or under restricted diets and overcoming linguistic and other human cultural barriers that prevent accurate communication of diet with self-reporting. Applying DNA metabarcoding to a wider range of human cohorts should be used to determine the utility of the approach for identifying dietary signals diagnostic or causal of various human diseases. Altogether, DNA metabarcoding has become increasingly common in environmental biology (7), and we believe that future applications and refinements of the approach described here could be valuable in studies of human nutrition and health. In conjunction with applying other molecular approaches to human samples, such as microscopy, stable isotope probing, and multi-omics techniques (37–39), a more complete picture of human diets is possible.

## MATERIALS AND METHODS

### Experimental diet study samples and metadata.

Fecal DNA samples were obtained from a previous experimental study on the effects of short-term dietary interventions on the microbiota (19). Analyses were determined to be exempt by the Duke Health Institutional Review Board (Pro00100567). Samples originated from 11 study participants who collected feces each day during 4 days of baseline analysis, 5 days of a plant-based diet, and 6 days of washout and then again for 4 days of baseline, 5 days of an animal-based diet, and 6 days of washout (see Fig. S1 in the supplemental material). The plant-based diet was composed of selected grains, legumes, fruits, and vegetables; the animal-based diet was composed of prepared meats, eggs, and cheeses (Table S1). On both diet arms of the experiment, participants were instructed to eat only study-provided meals and snacks or allowable beverages (water or unsweetened tea for both diets; coffee was allowed on the animal-based diet). They were also allowed to add one salt packet per meal, if desired for taste. Participants could eat unlimited amounts of the provided foods. Participants ate freely during the baseline and washout periods. Across all study days, participants kept daily diet diaries that recorded the quantity and makeup of their unconstrained diets during the baseline/washout periods and, similarly, the quantity and type of the prepared foods they chose to eat during the experimental diet arms. During both free-feeding and experimental diet arms, participants consumed a mix of both cooked and uncooked ingredients, but the preparation method was not always recorded. Rapid and reproducible changes in gut microbiota community structure, gene expression, and metabolism were detected across study participants during diet arms (19), which suggested that participants complied with study diet designs.

10.1128/mSystems.00458-19.2 TABLE S1 Ingredients included in plant and animal arms of dietary interventions. Plant ingredients noted in diet logs during noninterventional periods. Presence in the database and success in detection by metabarcoding are noted for each. Download Table S1, XLSX file, 0.01 MB. Copyright © 2019 Reese et al. 2019 Reese et al. This content is distributed under the terms of the Creative Commons Attribution 4.0 International license.

Samples were selected for plant DNA metabarcoding from the ends of the baseline period, experimental interventions, and washout periods (*n* = 54 fecal samples; Fig. S1). One participant did not participate in each arm of the experiment, and DNA was no longer available for some participants at certain time points, but we were able to include at least 9 participants from each diet-arm grouping. Diet-diary data were coded from diary entries on the day prior to fecal sample collection. DNA was extracted using a PowerSoil DNA extraction kit (MoBio) and then stored frozen as part of the original study. Data describing gut microbial composition and one measure of microbial function (short-chain fatty acid concentration) were also drawn from the work of David et al. (19). In short, microbial community composition was determined by 16S rRNA gene amplicon sequencing with the Illumina platform. Short-chain fatty acid concentrations were measured with gas chromatography.

### DNA metabarcoding sequencing and processing.

We used the P6 loop of the chloroplast *trn*L (UAA) intron (*trn*L-P6), which is a broad-spectrum marker useful for DNA metabarcoding of plant species, with published primers (7) and established laboratory protocols (8). Briefly, the *trn*L-P6 locus was amplified with molecular identification (MID) tags to enable pooling and demultiplexing. Pooled amplicons were assembled into a library using the Apollo 324 NGS Library Prep system and PrepX DNA kit (WaferGen, CA), which included DNA end-repairing, A-tailing, adapter ligation, and limited amplification before Illumina barcodes were ligated to the pool for sequencing on an Illumina HiSeq 2500 Rapid Flowcell at Princeton University’s Lewis Sigler Institute as single-end 170-nucleotide (nt) reads.

We compiled a reference database comprising the *trn*L-P6 sequences of commonly consumed plant species. To obtain reference sequences, we compiled a list of scientific names from 86 domesticated plant taxa and queried GenBank for records matching “*trn*L” and each of these genus- or species-level groups. A total of 4,688 sequences matching these search terms were downloaded from GenBank in October 2016, and we used the ecoPCR function from the obitools software (40) to search these records for the full-length *trn*L-P6 marker. In this search, we allowed for up to 4 mismatches to the same primers used in metabarcoding analyses and considered sequences spanning 9 to 300 bp in length. We retained reference sequences that were identifiable to genus level using the NCBI taxonomic database. A total of 185 unique sequences representing 2,162 GenBank accessions representing 72 species were obtained from this search for the full-length *trn*L-P6 reference sequence (Data Set S1). The number of sequences in the database exceeds the number of plant species considered in the search because some food species may be represented by multiple haplotypes or because they are represented by congeneric taxa. Based on this database, some common food items are difficult or impossible to distinguish genetically from close relatives despite readily apparent phenotypic differences that can be noted in diet logs (e.g., broccoli, Brussels sprouts, and cabbage [Brassica oleracea]; pumpkin and zucchini [Cucurbita pepo]; hot and bell peppers [Capsicum annuum]; citrus fruits [*Citrus* spp.]); others are phenotypically similar and called the same common name but are different species (e.g., berries that include members of the genera *Rubus*, *Vaccinium*, and *Fragaria* and various species of *Phaseolus* collectively referred to as “beans”). These genetic issues prevented us from identifying some metabarcoding-derived sequences to the species level, and the lexical issues prevented us from identifying some self-reported foods to the species level. Taxa that could not be distinguished by sequence or by name were combined at a higher taxonomic level, and the corresponding entries in diet logs were similarly combined for accurate comparison. These changes affected the taxonomic assignment of 26 unique *trn*L-P6 sequences from the metabarcoding analysis (Tables S2 and S3), and the resulting taxonomic classification was used in all subsequent analyses. In some cases, sequences were unavailable in GenBank or their species-level identifications were deemed uncertain. This affected a few plants found in participant diet logs, including various spices and cranberry, and these taxa were excluded from downstream analyses for both metabarcoding and diet-log analyses (Table S1).

10.1128/mSystems.00458-19.3 TABLE S2 Description of taxa in the food-plant DNA reference library. For each unique *trn*L-P6 sequence, we list a representative GenBank accession number, the level of resolution (genus or species) that each sequence represents in this database, and the family and genus affiliation of each unique sequence. The set of species in the database represented by each sequence is listed together with the number of downloaded GenBank accessions that share the identical *trn*L-P6 sequence (*n* = 1 to 421; median = 2) and the number of unique species names attributed to those accessions (*n* = 1 to 94; median = 1). We also list the most relevant scientific and common names for each species or set of species. In some cases, the relevant scientific name of the domestic species did not appear in any of the GenBank accessions (e.g., kiwi, *Actinidia deliciosa*, was not present in GenBank, but a search for *Actinidia* spp. yielded two unique *trn*L-P6 sequences from 42 accessions representing 23 species). In other cases, the same scientific name of a domestic species matched multiple GenBank accessions represented by more than one unique *trn*L-P6 sequence (e.g., onion, *Allium cepa*). Finally, different parts or varieties of the same plant species can be consumed under multiple common names (e.g., cabbage, broccoli, cauliflower, kale, Brussels sprouts, collard greens, savoy, and kohlrabi are all *Brassica oleracea*). Thus, the relevant scientific and common names attributed to each *trn*L-P6 sequence in this reference library serve as heuristics to facilitate identification of food DNA based on the best available sequences from GenBank. The exact search string used to build this database is as follows: “trnL” AND (“Abelmoschus esculentus”[Organism] OR “Actinidia”[Organism] OR “Allium cepa”[Organism] OR “Allium sativum”[Organism] OR “Ananas comosus”[Organism] OR “Apium graveolens”[Organism] OR “Arachis hypogaea”[Organism] OR “Asparagus officinalis”[Organism] OR “Avena sativa”[Organism] OR “Brassica juncea”[Organism] OR “Brassica oleracea”[Organism] OR “Brassica rapa rapa”[Organism] OR “Camellia sinensis”[Organism] OR “Capsicum annuum”[Organism] OR “Carica papaya”[Organism] OR “Carya illinoinensis”[Organism] OR “Cichorium endivia”[Organism] OR “Citrullus lanatus”[Organism] OR “Citrus limon”[Organism] OR “Citrus paradisi”[Organism] OR “Citrus sinensis”[Organism] OR “Citrus tangerina”[Organism] OR “Coffea”[Organism] OR “Corylus avellana”[Organism] OR “Cucumis melo”[Organism] OR “Cucumis sativus”[Organism] OR “Cucurbita pepo”[Organism] OR “Cucurbita”[Organism] OR “Cynara cardunculus”[Organism] OR “Daucus carota”[Organism] OR “Fragaria ananassa”[Organism] OR “Hordeum vulgare”[Organism] OR “Ipomoea batatas”[Organism] OR “Juglans regia”[Organism] OR “Lactuca sativa”[Organism] OR “Lactuca sativa longifolia”[Organism] OR “Macadamia integrifolia”[Organism] OR “Macadamia jansenii”[Organism] OR “Macadamia ternifolia”[Organism] OR “Macadamia tetraphylla”[Organism] OR “Malus domestica”[Organism] OR “Mangifera indica”[Organism] OR “Musa acuminata”[Organism] OR “Olea europaea”[Organism] OR “Oryza sativa”[Organism] OR “Persea americana”[Organism] OR “Phaseolus lunatus”[Organism] OR “Phaseolus vulgaris”[Organism] OR “Pistacia vera”[Organism] OR “Pisum sativum”[Organism] OR “Prunus”[Organism] OR “Prunus armeniaca”[Organism] OR “Prunus avium”[Organism] OR “Prunus dulcis”[Organism] OR “Prunus persica”[Organism] OR “Pyrus”[Organism] OR “Raphanus sativus”[Organism] OR “Rubus idaeus”[Organism] OR “Rubus strigosus”[Organism] OR “Rubus idaeus subsp. strigosus”[Organism] OR “Saccharata rugosa”[Organism] OR “Secale cereale”[Organism] OR “Solanum lycopersicum”[Organism] OR “Solanum melongena”[Organism] OR “Solanum tuberosum”[Organism] OR “Spinacia oleracea”[Organism] OR “Theobroma cacao”[Organism] OR “Citrus aurantifolia”[Organism] OR “Triticum aestivum”[Organism] OR “Vaccinium”[Organism] OR “Vitis”[Organism] OR “Zea mays”[Organism] OR “Chenopodium quinoa”[Organism] OR “Colocasia esculenta”[Organism] OR “Coriandrum sativum”[Organism] OR “Durio”[Organism] OR “Fagopyrum esculentum”[Organism] OR “Helianthus annuus”[Organism] OR “Linum usitatissimum”[Organism] OR “Vaccinium oxycoccos”[Organism] OR “Vaccinium macrocarpon”[Organism] OR “Phoenix dactylifera”[Organism] OR “Salvia hispanica”[Organism] OR “Lens culinaris”[Organism] OR “Glycine max”[Organism] OR “Salvia”[Organism]). Download Table S2, XLSX file, 0.02 MB. Copyright © 2019 Reese et al. 2019 Reese et al. This content is distributed under the terms of the Creative Commons Attribution 4.0 International license.

10.1128/mSystems.00458-19.4 TABLE S3 Summary of modified taxonomic assignments for *trn*L-P6 sequences included in the reference database and identified from stool samples by DNA metabarcoding. Sequence ID, identifier for P6 sequence assigned by obitools; Original name/Original taxid, assignment of reference sequence in NCBI Entrez Taxonomy; Updated name/Updated taxid, assignment based on curation in response to genetic and lexical issues as summarized in Materials and Methods. Download Table S3, XLSX file, 0.01 MB. Copyright © 2019 Reese et al. 2019 Reese et al. This content is distributed under the terms of the Creative Commons Attribution 4.0 International license.

10.1128/mSystems.00458-19.5 TABLE S4 Summary statistics for individual taxa measured with metabarcoding. Taxid, taxonomic identifier by NCBI Entrez Taxonomy; Taxonomic level, the level to which the taxon can be specified (family, genus, or species); Name, Latin name of plant; Number unique P6 sequences, the number of distinct sequences generated by DNA metabarcoding that were mapped to the plant taxon by obitools; Number samples, the number of samples in which the plant taxon was detected; Number reads, the total number of sequence reads assigned to the taxon across all samples; FPR, false-positive rate; FNR, false-negative rate. Note that FNR is NA if the plant taxon was never recorded as consumed in diet records, and thus the denominator in its calculation evaluates to 0. Some plants have an FPR of 1 and an FNR of 0; this occurs when the plant was detected in every sequenced stool sample, and thus, there are no false or true negatives to include in calculation of either value, reducing them to 1 and 0, respectively. Download Table S4, XLSX file, 0.01 MB. Copyright © 2019 Reese et al. 2019 Reese et al. This content is distributed under the terms of the Creative Commons Attribution 4.0 International license.

10.1128/mSystems.00458-19.6 TABLE S5 Recall, precision, and F-measure varied depending on diet arm and thresholding. Varied analysis parameters are arrayed across the columns, while subsets of samples are arrayed in rows. Note that a taxonomic level of “all” indicates that all plant taxa were included side by side in the calculation, resulting in a mix of taxa at the family, genus, and species levels. Otherwise, the taxa were restricted and/or summarized to meet the listed taxonomic level, and their resulting count was recorded under “unique taxa in comparison.” Download Table S5, XLSX file, 0.01 MB. Copyright © 2019 Reese et al. 2019 Reese et al. This content is distributed under the terms of the Creative Commons Attribution 4.0 International license.

10.1128/mSystems.00458-19.7 DATA SET S1 Reference database comprising the *trn*L-P6 sequences of plant species commonly consumed by humans. Download Data Set S1, TXT file, 0.1 MB. Copyright © 2019 Reese et al. 2019 Reese et al. This content is distributed under the terms of the Creative Commons Attribution 4.0 International license.

The fecal DNA sequences were demultiplexed and identified through comparison to the reference database. Demultiplexing, identification, and quality controls were performed using obitools software (40). At this stage, we removed sequences with >2 mismatches to the primers, sequences with Illumina fastq quality scores averaging ≤32 across the length of the *trn*L-P6 sequence, sequences that contained any ambiguous base calls, and sequences that were <9 bp. We tallied identical sequences in the remaining data set and dropped those that occurred <10 times across all samples that were included in the data set (including controls, extraction blanks, and dietary samples that were subsequently dropped from analysis). A data set of 21,325 unique sequences (2,899,718 total sequence reads) was produced, and only sequences with 100% match identity to a food-plant sequence in the reference database were retained for further analyses (*n* = 78 perfect matches in comparison to the 185 unique *trn*L-P6 sequences in the database).

### Analyses.

The DNA metabarcoding results were benchmarked for their precision and recall compared to recorded diet. Our benchmarking procedure required assumptions about the completeness of diet records, and because these are known to have frequent inaccuracies (2–4), our results may best be interpreted as estimates of precision and recall. We assumed that omission of foods from diet diaries due to memory lapses, selective reporting, or intake of prepared or processed foods in which not all ingredients were known to the consumer was more likely than the erroneous reporting of a food that was not in fact consumed. Thus, we prioritized metrics that make comparisons between metabarcoding and foods reported as present (rather than absent) in diet diaries. We calculated (i) recall (also called sensitivity), defined as the percentage of foods in diet diaries that were also detected by DNA metabarcoding, and (ii) precision (also called positive predictive value), defined as the percentage of plant taxa detected by DNA metabarcoding that were also recorded in diet diaries. These calculations were performed by comparing diet records that coded a plant taxon as present or absent to the metabarcode read counts that corresponded to the same plant taxon. Because there is an inverse relationship between precision and recall, we also calculated the F-measure, which represents the harmonic mean of precision and recall and ranges from 0 (completely inaccurate detection) to 1 (perfect precision and recall).

For calculation of precision and recall at different taxonomic levels, species were collapsed to shared genera and genera were collapsed to shared families by summing read counts (in the case of metabarcoding data) or by combining binary presence/absence data using an “OR” operator (in the case of reported consumption of a plant taxon in the diet). We repeated this calculation by applying common thresholds of sequence relative read abundance required to infer the “presence” of a plant within a sample (i.e., >0%, 1%, and 5%).

We performed Mantel tests to compare the diets captured by metabarcoding and participant reporting as well as to compare diet summaries and gut microbial composition and functioning. Metabarcoding, microbial composition, and short-chain fatty acid data were processed using the abundance-weighted Bray-Curtis dissimilarity, whereas diet diary data were analyzed only as presence/absence (Jaccard index). Analyses were conducted on each experimental window separately (baseline, plant diet intervention, plant diet washout, and animal diet washout) to exclude multiple measurements of the same individual. Bonferroni corrections were applied to address multiple-hypothesis testing. To determine if metabarcoding and/or participant recording reflected the effect of the experimental diet treatments (free eating, animal diet, or plant diet) we performed permutational multivariate analysis of variance (PERMANOVA). Tests were performed with the vegan package (41) in R (version 3.3) (42).

### Data availability.

Sequencing data acquired for this study are available through the European Nucleotide Archive under accession number PRJEB34336. The reference sequences are available in Data Set S1.
