# Supplementary figures and images for: Using DNA Metabarcoding To Evaluate the Plant Component of Human Diets: a Proof of Concept
Source: mSystems. 2019 Oct 8;4(5):e00458-19. doi: 10.1128/mSystems.00458-19 (PMC6787566; doi:10.1128/mSystems.00458-19)

*Date*

Baseline

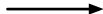

Plant-based diet

Washout

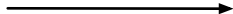

N=2

N=1

N=7

N=1

N=1

N=6

-4

-3

-2

-1

0

1

2

3

4

5

6

7

8

9

10

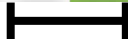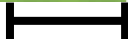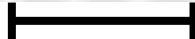

*Date*

Baseline

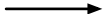

Animal-based diet

Washout

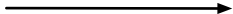

N=2

N=1

N=1

N=2

N=2

-4

-3

-2

-1

0

1

2

3

4

5

6

7

8

9

10

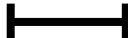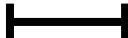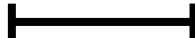

Supplement: FIG S1 [file mSystems.00458-19-sf001.pdf]
